# Automated hippocampal segmentation algorithms evaluated in stroke patients

**DOI:** 10.1038/s41598-023-38833-z

**Published:** 2023-07-20

**Authors:** Marianne Schell, Martha Foltyn-Dumitru, Martin Bendszus, Philipp Vollmuth

**Affiliations:** Department of Neuroradiology, Heidelberg University Hospital, Im Neuenheimer Feld 400, 69120 Heidelberg, Germany

**Keywords:** Brain, Stroke, Medical research

## Abstract

Deep learning segmentation algorithms can produce reproducible results in a matter of seconds. However, their application to more complex datasets is uncertain and may fail in the presence of severe structural abnormalities—such as those commonly seen in stroke patients. In this investigation, six recent, deep learning-based hippocampal segmentation algorithms were tested on 641 stroke patients of a multicentric, open-source dataset ATLAS 2.0. The comparisons of the volumes showed that the methods are not interchangeable with concordance correlation coefficients from 0.266 to 0.816. While the segmentation algorithms demonstrated an overall good performance (volumetric similarity [VS] 0.816 to 0.972, DICE score 0.786 to 0.921, and Hausdorff distance [HD] 2.69 to 6.34), no single out-performing algorithm was identified: FastSurfer performed best in VS, QuickNat in DICE and average HD, and Hippodeep in HD. Segmentation performance was significantly lower for ipsilesional segmentation, with a decrease in performance as a function of lesion size due to the pathology-based domain shift. Only QuickNat showed a more robust performance in volumetric similarity. Even though there are many pre-trained segmentation methods, it is important to be aware of the possible decrease in performance for the segmentation results on the lesion side due to the pathology-based domain shift. The segmentation algorithm should be selected based on the research question and the evaluation parameter needed. More research is needed to improve current hippocampal segmentation methods.

## Introduction

Structural and functional changes in the hippocampus can predict cognitive decline as a key element for patients’ quality of life^1–3^. As a result, the volumetry of brain magnetic resonance imaging (MRI) data is increasingly recognized and used as a biomarker for the early detection and diagnosis of dementia. For Alzheimer’s patients, cross-sectional measurements of brain atrophy patterns are represented in recent consensus diagnostic guidelines as important supporting features^4^. Although the hippocampus is rarely directly involved in ischemic strokes^5^, the risk of dementia is significantly increased, with approximately one-third of patients experiencing transient or permanent cognitive impairment after stroke^6–8^. Current research often focuses on physical disabilities, while the cognitive aspects are often neglected^9–11^.

Large-scale analysis will help to identify neuroimaging biomarkers for early detection and intervention of post-stroke dementia. However, inferring functionality often requires not only the hippocampal volume but accurate delineation for subsequent extraction of functional parameters such as ADC values or perfusion parameters. Reproducible segmentation of the hippocampus can help to study in vivo and understand the underlying functional and structural hippocampal changes causing this cognitive decline in post-stroke dementia.

Segmenting the hippocampus can be challenging due to the small or absent signal gradients between the structure and adjacent regions. To date, manual segmentation by a radiologist is still considered the gold standard among neuroanatomical experts. Unfortunately, it requires expertise, is very time-consuming^12^, and carries a high risk of intra- and interobserver variability, resulting in a low reproducibility^13^.

Automated segmentation methods are proposed as a reliable alternative to human manual tracing. While traditional atlas-based approaches (e.g., FreeSurfer segmentation^14,15^) can generate precise segmentations, they may not be effective for patients with significant structural changes, such as stroke lesions^16^.

Recently, more time-efficient deep learning-based approaches were increasingly applied in the domain. Training of these algorithms usually requires pre-annotated datasets as ground truth segmentations for supervised learning. Like manual segmentation, time-consuming delineation can bias the learning process by the subjective decisions of the rater. Furthermore, numerous pre-trained, open-source deep learning-based algorithms are readily available, allowing for rapid hippocampal segmentation of new, large-scale datasets without requiring prior training^17–22^. Unfortunately, these networks are mainly trained on healthy volunteers or special patient groups such as Alzheimer's patients and are not explicitly designed to account for other brain disorders. Large brain lesions common to stroke patients represent a domain shift for these pre-trained segmentation networks^23,24^, which may cause a significant drop in the segmentation performance.

Not only is the training of these networks based on pre-annotated datasets, but the evaluation of segmentation results is traditionally based on agreement measures with reference segmentation. However, most large datasets lack this reference ‘ground truth’ segmentation, making conventional performance measurement evaluation impossible. For this work, we generated a "virtual" ground truth segmentation based on a consensus method using simultaneous truth and performance level estimation (STAPLE) algorithm. Omitting manual pre-labeled data will lead to more objective and reproducible results.

In this study, we explored the generalizability of recent, pretrained, open-source, deep learning-based hippocampal segmentation networks. We introduced a domain shift by changing the population to a new and unseen dataset with chronic stroke lesions to test the cross-domain transferability.

The aim was to analyze the segmentation performance with common evaluation metrics based on an agreement approach: (1) by ranking the algorithms to a virtually generated ground truth segmentation using the STAPLE algorithms and (2) to visualize (dis)similarities in a pairwise comparison using mean values with a metric multidimensional scaling approach. (3) In a subgroup analysis we further analyzed whether the presence of the stroke lesion negatively impacted the segmentation performance. The robustness of the segmentation results was evaluated by the correlation of evaluation metrics and stroke volume.

## Results

### Dataset

We included n = 641/655 patients (97.86%) from n = 33 institutes. The remaining n = 14 patients (2.14%) were excluded due to movement artifacts (n = 3), due to inadequate image resolution (n = 7), and due to hippocampal involvement of the stroke lesion (n = 4). An overview of the ATLAS dataset can be found in Table 1.

**Table 1:** Overview table of ATLAS 2.0 dataset with original training dataset, the final dataset used for analysis after excluding n = 14 patients and the subset used for the manual tracing and FreeSurfer segmentation, controlled for the stroke volume, Kolmogorov–Smirnov test: D = 0.152, p = 0.523.

Variables	Original training dataset	Final dataset	Manual tracing subset
	(N = 655)	(N = 641)	(N = 30)
Field strength of scanner			
1.5 T	52 (8%)	51 (8%)	3 (10%)
3 T	603 (92%)	590 (92%)	27 (90%)
Vendor			
GE	204 (31%)	204 (32%)	7 (23%)
Philips	96 (15%)	94 (15%)	5 (17%)
Siemens	355 (54%)	343 (53%)	18 (60%)
Volume of voxel			
Mean ± sd	1 ± 0.38	1 ± 0.38	0.91 ± 0.24
Stroke hemisphere			
Right	289 (44%)	286 (45%)	16 (53%)
Left	294 (45%)	283 (44%)	14 (47%)
Other	72 (11%)	72 (11%)	NA
Volume of stroke in mm^3^			
Median (Q1–Q3)	3775 (895–26,618)	3727 (891–25,816)	5066 (1107–39,997)

In total the automatic segmentation failed for n = 53 cases from n = 48 patients, n = 8 for the e2dhipseg (n = 5 on the ipsilesional side), and n = 45 for the HippMapp3r algorithms (n = 32 on the ipsilesional side). All other algorithms showed a segmentation success rate of 100%. No erroneous STAPLE masks were detected after the visual inspection.

### Volumes of segmentation and STAPLE masks

**Figure 1:**
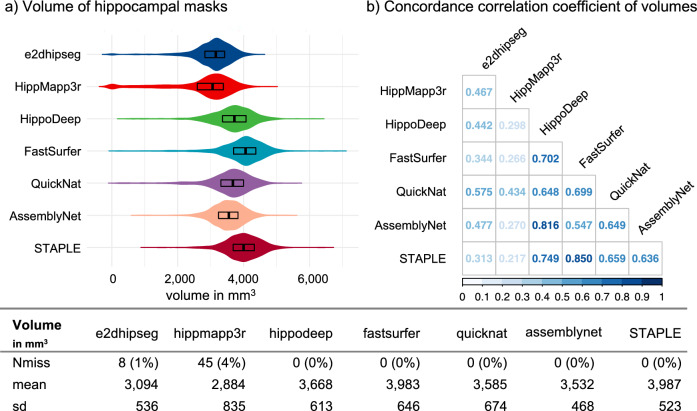
Volumetric analysis of hippocampal masks. (**a**) Violin plots for the segmentation algorithms and STAPLE ground truth, (**b**) concordance correlation coefficients for the segmented volumes in all comparisons. The bottom table for the summary of mask volumes, with the number of missing segmentations, mean, and standard deviation of the extracted volumes.

Figure 1 depicts the hippocampal volumes as well as the concordance correlation coefficients of each segmentation algorithm with the virtual generated STAPLE ground truth. FastSurfer segmentation showed an excellent agreement in volume with the STAPLE ground truth and a concordance correlation coefficient of 0.85. Three out of six comparisons revealed a good agreement (Hippodeep, QuickNat, and AssemblyNet) and the remaining two algorithms with a fair agreement.

The interpretation of all pairwise comparisons allows conclusions about agreement across methods. Only the comparison between Hippodeep and the AssemblyNet segmentations revealed an excellent agreement with a CCC of 0.815. In total seven (33.3%) pairs out of all 21 comparisons had a good agreement, six (28.6%) with a poor agreement and six (28.6%) with a moderate agreement (in detail, see Fig. 1b).

### Evaluation metrics of segmentation results

Results for the evaluation metrics of the segmentation results in relation to the virtual generated STAPLE ground truth can be found in Table 2. Segmentation methods achieved good performances compared to the STAPLE segmentation with mean values between 0.816 and 0.953 for volumetric similarity, 0.854 and 0.921 for DICE score, and 2.69 and 6.34 for Hausdorff distance.

**Table 2:** Summary of the evaluation metrics for the segmentation algorithms in relation to the STAPLE masks.

Algorithms	Failed (n)	Volumetric similarity (mean ± std)	DICE score (mean ± std)	Average hausdorff distance (mean ± std)	Hausdorff distance 95 (mean ± std)
e2dhipseg	8 (1%)	0.869 ± 0.076	0.854 ± 0.074	0.271 ± 2.63	3.83 ± 3.75
hippmapp3r	45 (4%)	0.816 ± 0.166	0.786 ± 0.186	156 ± 4270	6.34 ± 9.56
hippodeep	**0 (0%)**	0.953 ± 0.056	0.909 ± 0.056	0.114 ± 0.204	**2.69 ± 1.76**
fastsurfer	**0 (0%)**	**0.972 ± 0.056**	0.910 ± 0.058	0.112 ± 0.121	3.60 ± 2.11
quicknat	**0 (0%)**	0.939 ± 0.087	**0.921 ± 0.088**	**0.101 ± 0.250**	3.16 ± 3.23
assemblynet	**0 (0%)**	0.938 ± 0.027	0.887 ± 0.026	0.126 ± 0.041	3.32 ± 1.21

The best value of each metric is in bold.

The best-performing algorithm in volumetric similarity was FastSurfer (mean = 0.910, sd = 0.058). FastSurfer had a statistically significantly better performance than the second-best performing algorithm Hippodeep with a mean difference of 0.019, 95% CI [0.016, 0.022], t(1281) = 12.112, p < 0.0001.

The best-performing algorithm in DICE score was QuickNat (mean = 0.939, sd = 0.056) with statistically significant better performance than the second-best performing algorithm FastSurfer with a mean difference of 0.011, 95% CI [0.006, 0.015], t(1281) = 4.55, p-value < 0.0001.

The best-performing algorithm in HD95 was Hippodeep (mean = 2.69, sd = 1.76) with statistically significantly better performance than the second-best performing algorithm QuickNat with a mean difference of − 0.47, 95% CI [− 0.65, − 0.30], t(1281) = − 5.31, p-value < 0.0001.

For the average HD there was no significant difference between the two best-performing algorithms QuickNat and FastSurfer with a mean difference of − 0.01, 95% CI [− 0.03, − 0.001], t(1281) = − 1.07, p-value 0.082.

**Figure 2:**
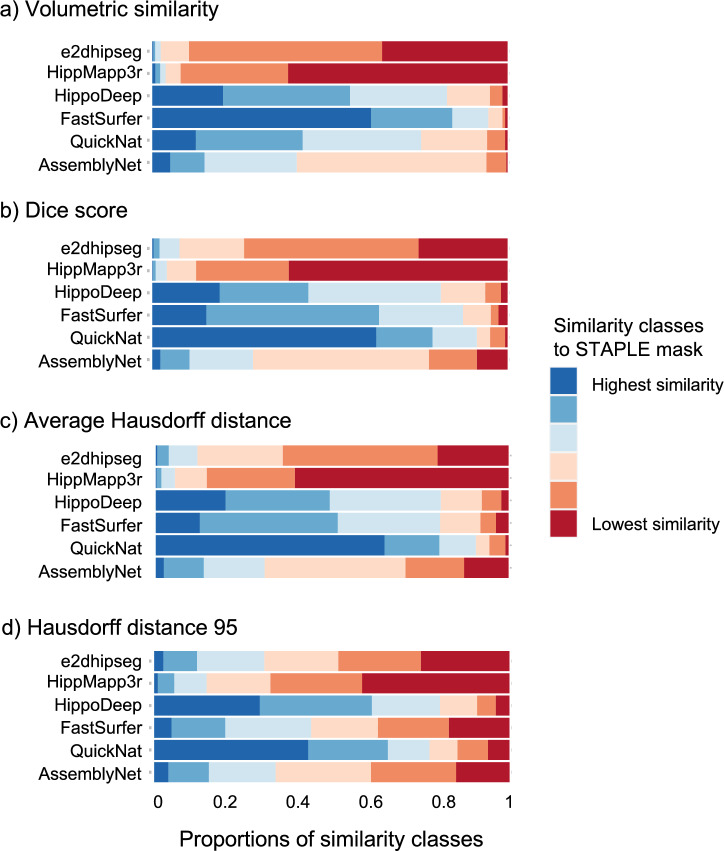
Instance-based similarity classification for similarity ranks to STAPLE masks. Equal values were both assigned to the inferior category to avoid additional intermediary categories. Bark blue color with the highest similarity to STAPLE mask, red color with the lowest similarity.

Instance-based classification revealed Hippodeep, FastSurfer, and QuickNat as the three most similar algorithms to the STAPLE masks in volumetric similarity, DICE score, and average HD (Fig. 2). In general, DICE scores and average HD revealed similar ranking distributions (Fig. 2b,c) showing QuickNat as the most similar segmentation to the STAPLE mask. The analysis of the HD95 showed a more heterogeneous result with the lowest similarities to the STAPLE masks for 41.5% of HippMapp3r masks, 24.9% of e2hipseg masks, 17.1% of FastSurfer masks, 15.1% of AssemblyNet masks, 6.1% of QuickNat masks, and 3.9% of Hippodeep masks, all shown as dark red bars in Fig. 2d.

**Figure 3:**
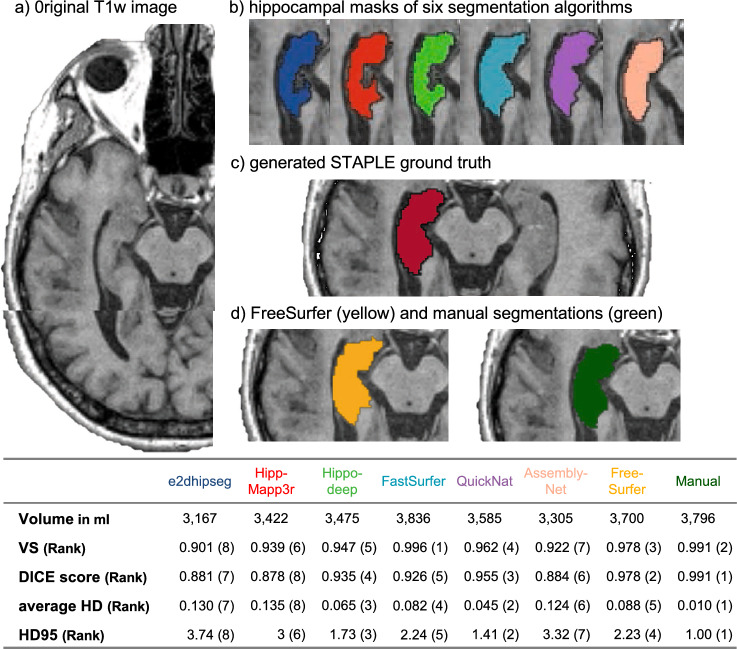
Representative example of segmentation results. (**a**) With a cropped axial slice of the original T1 weighted image, (**b**) the results of the hippocampal segmentation algorithms on the axial slice (blue for e2dhipseg, red for HippMapp3r, green for Hippodeep, light blue for FastSurfer, purple for QuickNat, and tan for AssemblyNet), (**c**) for the generated virtual STAPLE ground truth segmentation, (**d**) Manual and FreeSurfer segmentations. Bottom Table with the extracted volume and evaluation metrics compared to STAPLE mask, corresponding similarity classes in parenthesis.

A representative example of the segmentation results can be seen in Fig. 3. Albeit FastSurfer had the highest volumetric similarity to the STAPLE masks, the result did not reveal the highest DICE score compared, unveiling an undersegmented hippocampal head and oversegmented tail compared to the STAPLE mask (see 3D rendering, Supplementary Fig. S1).

**Figure 4:**
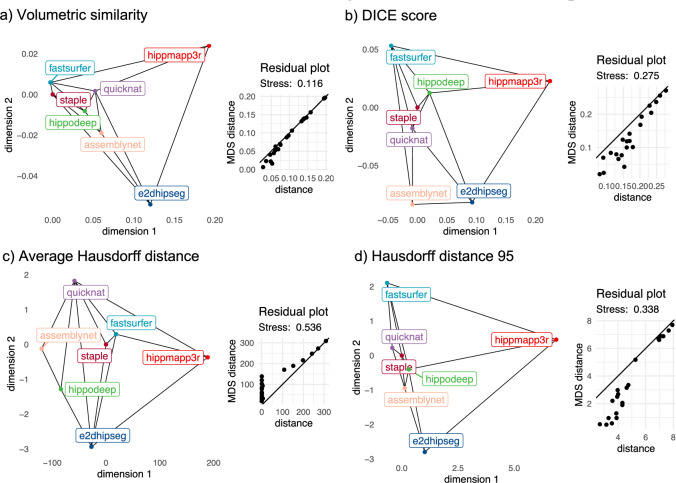
Multidimensional scaling maps and residual plots of the mean distance matrix of (**a**) volumetric similarity, (**b**) DICE score, (**c**) average HD, and (**d**) HD95. The proximity of the two methods can be interpreted as similarity, the smaller the distance the more similar are the methods on average.

Figure 4 shows the MDS maps for volumetric similarity, DICE score, HD95, and AVGHD based on the mean values and the residual plots. The maps allow interpreting not only the mean (dis)similarity between the algorithms and the STAPLE mask but also the similarity of two segmentation algorithms by the proximity of these algorithms and the difference by distance. For example, the maps showed that the mean segmentation results of the e2dhipseg algorithm are more similar to the mean AssemblyNet segmentation masks than to the Hippodeep segmentation results (for all evaluation metrics).

Residual plots and the stress value provides the goodness-of-fit statistic of the MDS plots. The best fit was found for volumetric similarity. For the other two metrics, some short distances were underestimated in the MDS maps.

### Subgroup analyses for hemispheric stroke lesions

All algorithms revealed a smaller segmentation volume for the ipsilesional side compared with the opposite side (Supplementary Table S2 online).

The agreement analysis with the STAPLE segmentation masks showed a significant decrease in similarities in all three metrics (lower volumetric similarities and DICE score and higher Hausdorff distance) for HippMapp3r, Hippodeep, FastSurfer, and AssemblyNet (Supplementary Table S3 online). For the QuickNat segmentation results, the performance decrease was observed only for DICE score and Hausdorff distance.

**Figure 5:**
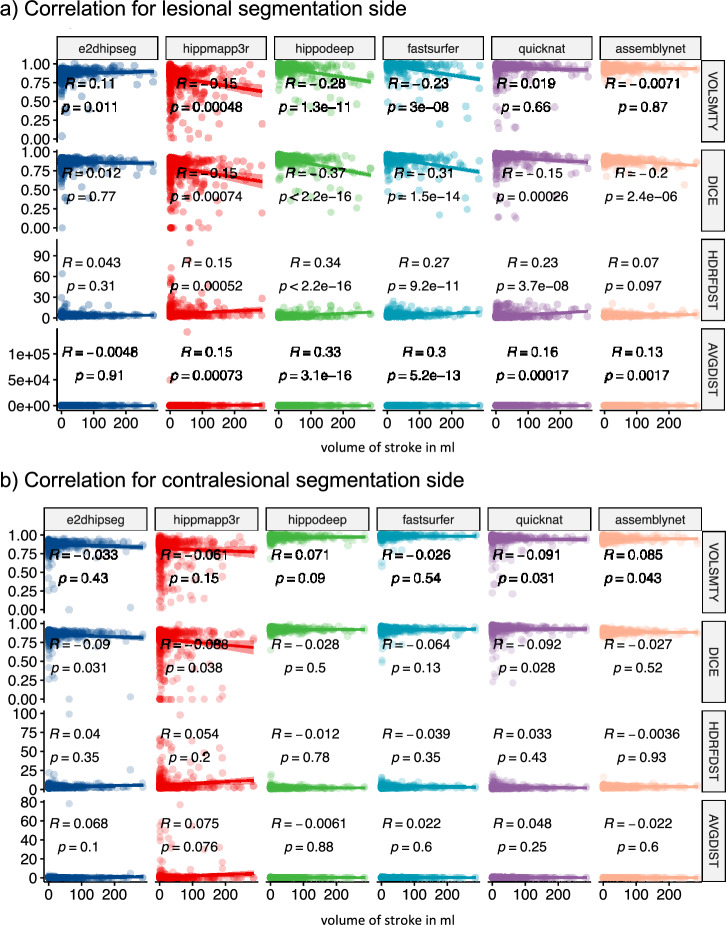
Spearman’s rank correlation of evaluation metrics and stroke volume for all segmentation algorithms. Uncorrected p-values.

Figure 5 shows Spearman’s rank correlation between the evaluation metrics and the stroke volume. Hippodeep and FastSurfer algorithms showed a moderate association of stroke volume and similarity to the STAPLE segmentation (absolute R values above 0.2) with a significantly increasing poorer performance with increasing lesion size. QuickNat segmentation showed this association for average HD and HD95 and to a lesser extent for the DICE score, no association was detected for volumetric similarity.

### Comparing with manual tracings in a small subset

In a smaller subset of 30 patients, the similarity to manual trancing was assessed. All results for the subgroup analysis with the manual trancing and FreeSurfer segmentation can be found in the supplement (Supplementary Table S4).

In 3 of 30 patients, the FreeSurfer algorithms failed to produce the segmentation on the lesion side (5%). For all mean evaluation metrics, the STAPLE masks showed the best results with a VS of 0.979 (0.016), DICE score of 0.979 (0.017), average HD of 0.023 (0.024), and HD95 of 2.11 (3.779), standard deviations in parenthesis. Supplementary Fig. S2 with the instance-based similarity classification to the manual segmentations. The rank distribution revealed a similar pattern to those obtained in the total dataset. Hippodeep and QuickNat with the best results among the deep learning-based methods. Interestingly, the volumetric similarity showed heterogeneity among the segmentation algorithms. FreeSurfer segmentation did not show high ranks regarding DICE, HD95, and average HD.

## Discussion

Reproducible and accurate image segmentation of in vivo magnetic resonance imaging is crucial for the reliable establishment of putative image biomarkers to improve the diagnostic and therapeutic decision-making processes^25–27^. In general, deep learning-based segmentation is not always more accurate, but more reproducible than human raters. Even though the application is simple and fast, the performance of these algorithms might be severely hindered by domain shift, which is rooted in differences between test data and training data used during algorithm development. Introducing new and unseen datasets to pre-trained algorithms may affect segmentation performance and should be tested before their implementation^28^.

This exploratory study investigated the cross-domain transferability of six pre-trained open-source hippocampal segmentation networks (e2dhipseg^19^, HippMapp3r^20^, Hippodeep^17^, FastSurferCNN^21^, QuickNat^18^ and AssemblyNet^22^) by exposing them to a dataset with structural signal alterations due to a chronic ischemic stroke lesion^29^. The applied and highly automated workflow enables an objective examination of (dis)similarity between the different segmentation results.

Even though the different segmentation algorithms were not developed for stroke patients, they showed a high success rate in segmenting the hippocampus with only a few missing segmentations. However, as expected, the volumetric results were not interchangeable (Fig. 1) and should be interpreted with caution^30^.

Stroke lesions rarely affect the hippocampus directly^5^, but all methods revealed a smaller volume on the side of the stroke lesion compared to the opposite side, a fact already known from the literature due to secondary neurodegeneration after the initial event^31–34^.

For this work, we used a statistical consensus method to generate a case-based virtual ground truth segmentation using the simultaneous truth and performance level estimation (STAPLE) algorithm. The usage of consensus methods—as a combination of several segmentation methods—improves the segmentation performance compared to a single method by compensating for the weaknesses of individual methods^35–38^.

In contrast to traditional methods (creating ground truth using manual segmentation), the methodological approach used in this work (creating a virtual GT mask by the STAPLE algorithm using only deep learning-based input segmentation) provides an increased reproducibility by avoiding subjective delineation or manual changes. Although the STAPLE algorithm is not entirely independent of the individual input segmentation masks, it allows for the assessment of the independent and individual contributions of each segmentation mask and method on an instance basis^38–40^. For a small subset, we could show that the STAPLE masks showed good results compared to manual tracing.

Segmentation methods achieved good performance compared to the STAPLE segmentation. Comparisons between the evaluation metrics did not identify a single algorithm that performed best. Instead, the algorithms showed inconsistency across different metrics, with three methods performing particularly well: FastSurfer^21^, QuickNat^18^, and Hippodeep^17^.

FastSurfer^21^ has demonstrated the best mean volumetric similarity and performs well in terms of CCC (concordance correlation coefficient). This algorithm is recommended for clinical applications where volumetric analysis alone is of primary interest. It could be employed in large-scale studies or clinical routine settings to reliably quantify hippocampal volume changes in stroke patients, which makes it a valuable tool for monitoring post-stroke dementia and disease progression. Interestingly, higher volumetric similarity does not necessarily indicate better overlap (DICE score), which suggests a misalignment of the segmentation results due to local over- and undersegmentation (e.g., the FastSurfer segmentation result in Fig. 3 and Supplementary Fig. 1).

QuickNat^18^ has demonstrated excellent performance in terms of DICE score, making it particularly suitable for applications that require precise anatomical delineation. Clinically, this algorithm could be utilized in studies focusing on the analysis of functional hippocampal changes (e.g., extraction perfusion values) and their correlation with cognitive decline in post-stroke patients.

Hippodeep^17^ has shown favorable performance in segmenting the hippocampus in stroke patients. While its volumetric similarity may not be as high as other algorithms, it excels in terms of mean Hausdorff distance (Table 2). This algorithm could be particularly valuable in clinical applications that require precise localization and boundary delineation of the hippocampus in stroke patients. For example, it could be employed in studies investigating the impact of stroke lesions on hippocampal shape or asymmetry, which could further increase our understanding of neurodegenerative processes in post-stroke patients.

Therefore, we advise choosing the most suitable segmentation method depending on the specific research question. Further post-processing steps, similar to those included in the e2dhipseg algorithm^19^, could enhance the distance measure by automatically eliminating small, distant voxels not connected to the two main volumes.

Acceptability from a clinical and scientific point of view should always be considered in the context of the research question. For example, oversegmentation can lead to a significant bias of extracted perfusion values, because neighboring anatomical structures, such as the choroid plexus have significantly higher perfusion values. Additionally, systematic bias, such as the oversegmentation of the smaller hippocampus on the lesion side, could pose a problem. However, analyzing this aspect is beyond the scope of the current project and warrants further investigation.

While deep learning-based segmentation methods have produced satisfactory results overall, the high-performing methods have demonstrated better performance and less variation when it comes to segmenting the contralateral side in comparison to the lesion side. This suggests that the algorithms may have limited generalization capability to the lesion side, likely due to their training. Additional evidence was provided by the result of the correlation analysis which showed a decreased segmentation performance with increasing lesion size only on the side of the lesion whereas these correlations were not evident for the segmentation masks on the opposite side (Fig. 5). The lack of robustness caused by the stroke lesion can be caused by the domain shift, but also by unadjusted or imprecise preprocessing steps, for example, due to internal registration processes. Further research is needed to optimize the segmentation results for the lesion side. Among the high-performing methods, the effect was the lowest for QuickNat segmentations with comparable performance in volumetric similarity, but differences in DICE score, suggesting a pronounced misalignment to the STAPLE mask on the lesion side.

To our knowledge, only one study^41^ examined automatic deep learning-based hippocampal segmentation in stroke patients showing better performance for the Hippodeep segmentation algorithm compared to the FreeSurfer segmentation, a traditional atlas-based method. The authors used a quality rating of volume by calculating over- and undersegmentation, but to correctly describe the performance of a segmentation method, several evaluation metrics are mandatory^42,43^. The use of various evaluation metrics is the prerequisite for morphological and functional analysis of anatomical structures, e.g., for the extraction of radiomic features^44^, where the exact delineation of a structure, considering the shape of the structure and its alignment, is essential.

Recently an updated version of FastSurfer was proposed, FastSurferVINN^45^, which might further improve the segmentation performance, but at the time of this publication, no code was available. Due to the public availability of the dataset, the analysis will be expendable for performance analysis of upcoming, novel segmentation algorithms.

Our work had some limitations. (1) Using a common agreement approach on data with an unknown ground truth segmentation assumes that the segmentation errors of the algorithms are uncorrelated. This assumption seems to be credible because all algorithms are based on different networks and trained on different datasets, which underlines the independence of these networks and minimizes systematic errors. (2) Common agreement methods can only approximate the “true” ground truth segmentation, its exact anatomical delineation is beyond the scope of this study and reserved for pathological or high-resolution imaging studies. Therefore, the use of the STAPLE mask can only determine the precision of a segmentation method, the true segmentation accuracy remains hidden. Given the higher variability of the segmentation results on the lesion side, the STAPLE maps could present reduced accuracy for the lesion side. However, all final STAPLE segmentation masks showed volumes in the expected physiological range without pronounced error detected by visual inspection and the expected reduced volume for the lesion side. (3) Due to the two-dimensionality of the MDS maps, visualizations showed a high-stress value. The use of additional dimensions would reduce the stress and thus increase the goodness-of-fit but hamper their interpretation. (4) Even if the general performance of the segmentation algorithm is good, no conclusions can be drawn about the case-specific performance, and individual segmentation masks may be erroneous. The prediction of case-specific confident values for the segmentation quality would help to determine the individual segmentation performance^46^. (5) For this work we used the training set of the ATLAS dataset, the sample size was not determined by a power analysis. Further, the use of only stroke patients may limit the generalizability of the finding to other patient populations. (6) We could not give detailed information on the processing time, but the corresponding requirements of the different segmentation algorithms are provided in the original publication.

The cross-domain transferability of six pretrained hippocampal segmentation networks was tested using a common agreement method. The analysis could not reveal one outperforming segmentation method, but rather various high-performing methods depending on the used evaluation metric. However, the overall performance of these methods on the lesion side showed higher variability and lack of robustness depending on the lesion size. Therefore, the best segmentation method should be chosen depending on the corresponding evaluation metric and the research question. For the application of ipsilesional hippocampal segmentation, additional training of new or existing segmentation networks with stroke datasets will further improve the cross-domain generalization of segmentation results. Currently, consensus methods can help optimize segmentation results on the lesion side.

In sum, accurate hippocampal segmentation will help reliably process large imaging datasets, facilitating large-scale stroke rehabilitation research with an appropriate sample size^47^. It will be ideal for automated integration in clinical routine to reveal subtle changes in the hippocampus and provide a basis for further research on post-stroke dementia^48^.

Further research is needed to optimize the quality of hippocampal segmentation in stroke patients.

## Methods

### Dataset

We used the Anatomical Tracing of Lesions after stroke (ATLAS) dataset release R2.0^29^. As described previously by Liew^29^, the dataset is derived from 33 cohorts worldwide. Only the training data was included, where manually annotated T1-weighted MRI sequences of chronic lesions after ischemic stroke were available, to investigate the dependence of hippocampal segmentation on the stroke lesion side, in total n = 655 patients. Each scan contained at least one lesion.

The sample size for the present analysis was determined by the availability of MRI data and was not derived from a power calculation.

An experienced observer (M.S. a board-certified radiologist) visually inspected all images for artifacts and hippocampal involvement. Further images with an insufficient resolution were removed, and a threshold was set to a voxel dimension of at least 1.5 mm. All T1-weighted images were reoriented to the standard orientation (fslreorient2std, FMRIB software library, http://fsl.fmrib.ox.ac.uk/fsl/fslwiki/FSL), followed by the segmentation process.

### Segmentation methods

Six, recent, open-source hippocampal segmentation algorithms were used:

- Three algorithms with hippocampal-only segmentation:
  - e2dhipseg including the recommended automatic orientation correction by rigid registration^19^ (https://github.com/MICLab-Unicamp/e2dhipseg)
  - HippMapp3r version 0.1.1^20^ (https://hippmapp3r.readthedocs.io/en/latest/install.html)
  - Hippodeep with the recommended Pytorch version^17^ (https://github.com/bthyreau/hippodeep_pytorch).
- Three algorithms for whole-brain anatomical segmentation (only the hippocampal masks were used for further processing):
  - FastSurfer^21^: FastSurferCNN: (segmentation only) docker image in GPU, release v1.0.1 (on 2 Apr, https://github.com/deep-mi/FastSurfer)
  - QuickNat^18^: available at https://github.com/ai-med/quickNAT_pytorch, and
  - AssemblyNet^22^, docker version 1.0.0 (https://github.com/volBrain/AssemblyNet).

All segmentation algorithms were used with the default or recommended parameter settings. Supplementary Table S1 contains general information regarding the computational requirements and processing times. We highly recommend consulting the related publications for a more comprehensive understanding of these details.

Each segmentation mask of the e2dhipseg algorithm contained only a common mask for both hippocampi. Additional postprocessing steps were added to divide the mask into the left and right hippocampus by splitting the MRI image in the mid-sagittal slice, followed by visual inspection and manual correction for cases with decentered or rotated images.

Hippodeep outputs a probability mask and was further thresholded at 0.5 as recommended^17^.

For deploying the FastSurfer and QuickNat algorithms an additional preprocessing step was needed, all T1w-images were standardized using the following command from FreeSurfer (mri_convert –conform), this re-samples the image to isotropic resolution (256 × 256 × 256) with some contrast enhancement. The resulting masks were back transferred to the original image space by rigid registration with 6 degrees of freedom using nearest neighbor interpolation to make the segmentation masks comparable to the other segmentation algorithms.

Further, a subgroup of n = 30 patients was selected using the FairSubset library^49^. Due to the possible bias due to the stroke volume, the “best” subset of patients with hemispheric stroke lesions was selected, controlled for the distribution of stroke volume. Manually segmented ground truth images were generated by M.F. a medical resident with 5 years’ experience. Additionally, a traditional segmentation approach was performed using FreeSurfer segmentation, version 7.1.1^14,15^. The resulting masks were back-transformed in the original patient space and compared to the manual trancing masks.

### Generation of ground truth

For each hippocampus a virtual ground truth image was generated using an expectation–maximization algorithm for simultaneous truth and performance level estimation (STAPLE)^39^, implemented in SimpleITK Release 2.0^50^ with a wrapper for Python (STAPLEImageFilter). The algorithm uses an iterative voting process to assign individual weights to each segmentation mask to compute probabilistic estimates of the "true" underlying segmentation. For each instance, the weights of the input segmentation masks are different. The binary STAPLE segmentation masks were generated using a probability threshold of 0.999. All STAPLE masks were visually inspected to detect erroneous estimations. No manual editing was applied at any stage of the process to ensure reproducibility.

### Performance evaluation

The following quantitative parameters were extracted to convert the segmentations into mineable data:

- *Volume* of the segmented hippocampus was extracted*,*
- *Segmentation success rate* (defined as completed segmentation and a hippocampal volume above zero) was estimated and
- *Common evaluation metrics*^51^ obtained using the EvaluateSegmentation tool^42^ available at http://github.com/codalab/EvaluateSegmentation for assessing inter-(dis)similarities between the six different segmentation algorithms and to the STAPLE ground-truth mask. The following four metrics were used:
  - Volumetric similarity (VS) to detect volume change,
  - DICE score as a spatial overlap-based metric to detect alignment errors,
  - average Hausdorff distance (AHD) and
  - Hausdorff distance to detect boundary errors at the 95th percentile (HD95) to overcome its sensitivity to outliers.

### Statistical analysis

All statistical analyses were performed with R version 4.2.1 (R Foundation for Statistical Computing, Vienna, Austria), all used R packages can be found in the supplement.

First, the resulting masks were compared with the STAPLE ground truth, segmentation volumes and performances were visualized. The similarity in the extracted volumes was analyzed using the concordance correlation coefficient (CCC). Concordance was classified as poor (0.00–0.20), fair (0.21–0.40), moderate (0.41–0.60), good (0.61–0.80), or excellent (0.81–1.00)^52^.

For each individual case and evaluation metric, segmentation results were classified into six categories for their similarity to the STAPLE mask. This analysis results in a case-by-case similarity ranking of the algorithms with respect to the STAPLE segmentation.

Further subgroup analyses were performed including only cases with hemispheric stroke lesions to compare the ipsi- and contralesional hippocampal segmentation result: (1) groups are compared with paired t-tests and (2) Spearman correlation to determine the relationship of evaluation metrics and stroke volume for both groups.

Finally, the mean (dis)similarities between the algorithms were visualized using metric multidimensional scaling (MDS) as a dimension reduction technique. For this purpose, the corresponding pairwise Euclidean distances were calculated for all segmentation pairs. The evaluation metrics for each pair were first averaged within a patient (across hemispheres) and then across all patients to generate mean evaluation metrics. For volumetric similarity and DICE score, all values were previously subtracted from 1 to obtain dissimilarity measures. The final distance matrices were determined using the cmdscale function^53^ to find the best-fitting two-dimensional representation of all mean segmentation algorithm results.

The resulting MDS maps visualized (dis)similarity between the mean segmentation results so that the distances among each pair of points correlate as best as possible to the dissimilarity between those two algorithms. Please note, the orientation of the MDS maps is entirely arbitrary and does not contain any information. For better comparison, the maps were centered on the STAPLE algorithm and rotated. Residual plots and stress values were depicted to display the goodness of fit to conform the configuration of the MDS map to the mean distance matrices.

To improve visualization, Delaunay triangulation was generated for the MDS maps to connect the most similar algorithms by an edge, using the tri.mesh function of the interp package 1.1-3^54^ implemented in R.

## Supplementary Information

Supplementary Information.

## Data Availability

Raw data in native space are available on the Archive of Data on Disability to Enable Policy and Research (ADDEP, 10.3886/ICPSR36684.v4). Requests to access the processed masks should be directed to M.S., marianne.schell@med.uni-heidelberg.de. Code and extracted values will be available at http://www.neuroAI-HD.org upon acceptance.
