# Supplementary material for: Automated hippocampal segmentation algorithms evaluated in stroke patients
Source: Sci Rep. 2023 Jul 20;13:11712. doi: 10.1038/s41598-023-38833-z (PMC10359355; doi:10.1038/s41598-023-38833-z)

Supplement to:

"Automated hippocampal segmentation algorithms evaluated in stroke patients."

Marianne Schell[1], Martha Foltyn-Dumitru[1], Martin Bendszus[1], Philipp Vollmuth[1]
[1]*Department of Neuroradiology, Heidelberg University Hospital, Heidelberg, Germany*

Used R version and R packages

- R version 4.2.1 (2022-06-23)
- attached packages:
    - corrplot 0.92
    - cowplot 1.1.1
    - DescrTab2 2.1.16
    - DescTools 0.99.47
    - dplyr 1.1.2
    - factoextra 1.0.7
    - fairsubset 1.0
    - forcats 0.5.2
    - ggforce 0.4.1
    - ggplot2 3.4.2
    - ggpubr 0.6.0
    - ggsci 2.9
    - here 1.0.1
    - interp 1.1-3
    - kableExtra 1.3.4
    - naniar 0.6.1
    - purrr 1.0.1
    - RColorBrewer 1.1-3
    - readr 2.1.2
    - rstatix 0.7.2
    - stringr 1.5.0
    - tibble 3.2.1
    - tidyr 1.5.0
    - tidyverse 1.3.2

Supplementary Table S1. *Segmentation algorithm with requirements and approximate processing time.*

Algorithms	Segmentation type	Recommended hardware	Software requirements (selection)	Approx. prediction time per volume
**e2dhipseg**	hippocampal-only	8GB of RAM<br>Ubuntu 16.04 or 18.04	Local Installation:<br>python3<br>pytorch<br>torchvision	5 min (CPU)<br>5 sec (GPU)
**hippmapp3r**	hippocampal-only	8GB of RAM<br>Linux, Windows, and MacOS.<br>No GPU necessary.	Local Installation:<br>python3.6<br>tensorflow<br>simpleitk<br>ANTs<br>c3d<br>Docker version available.	14 sec (GPU)
**hippodeep**	hippocampal-only	8GB of RAM<br>Linux CentOS 6.x/7.x, Ubuntu 18.04 and MacOS X 10.13,<br>No GPU and CUDA necessary.	Local Installation:<br>python3<br>pytorch	30 sec (GPU)
**fastsurfer**	whole-brain	8GB of RAM<br>Linux, Windows, and MacOS.<br>No GPU necessary.	Local installation:<br>python3<br>torch<br>torchvision<br>simpleitk<br>Docker version available.	14 min (CPU)<br>1 min (GPU)
**quicknat**	whole-brain	8GB of RAM<br>No GPU necessary.	Local installation:<br>python3<br>pytorch<br>torchvision<br>tensorflow	20 sec (GPU)
**assemblynet**	whole-brain	8GB of RAM<br>GNU/Linux or Windows 10/11 with WSL<br>No GPU necessary.	Docker version.	16 min (CPU)<br>14 min (GPU)

Supplementary Table S2. *Volume of ipsi- and contralesional segmentation. P-values from paired t-test, uncorrected.*

Algorithms	Ipsilesional volume (mean±std)	contralesional volume (mean±std)	p-value [95% CI]	[95% CI]
**e2dhipseg**	3,031 ± 630	3,089 ± 576	0.001	[-92, -23]
**hippmapp3r**	2,641 ± 1043	2,863 ± 912	<0.001	[-278, -164]
**hippodeep**	3,530 ± 699	3,778 ± 519	<0.001	[-298, -198]
**fastsurfer**	3,874 ± 744	4,062 ± 550	<0.001	[-237, -141]
**quicknat**	3,531 ± 708	3,619 ± 645	<0.001	[-125, -50]
**assemblynet**	3,458 ± 497	3,592 ± 443	<0.001	[-167, -101]
**STAPLE**	3,916 ± 563	4,037 ± 494	<0.001	[-157, -86]

Supplementary Table S3. *Within-patient comparison of segmentation results for volumetric similarity, DICE score, and Hausdorff distance in the subgroup with hemispheric stroke lesion, comparing ipsilesional (ipsil.) and contralesional (contral.) hippocampal segmentation masks in relation to the STAPLE ground truth segmentation. Uncorrected p-values from a paired t-test, 95% CI for the difference in means derived from the t-distribution. Patients with missing cases were removed.*

	**VS**		**DICE score**		**AHD**		**HD95**	
	Ipsil.	contral.	Ipsil.	contral.	Ipsil.	contral.	Ipsil.	contral.
**e2dhipseg**								
mean, std	0.873, 0.067	0.868, 0.065	0.856, 0.064	0.853, 0.064	0.17, 0.193	0.165, 0.157	3.70, 2.21	3.70, 1.74
p-value		0.012		0.099		0.569		0.994
[95% CI]		[0.001, 0.009]		[-0.001, 0.007]		[-0.011, 0.020]		[-0.18, 0.18]
**hippmapp3r**								
Mean, std	0.813, 0.164	0.832, 0.145	0.790, 0.168	0.808, 0.154	363, 6527	0.874, 5.49	5.98, 8.05	5.41, 7.78
p-value		<0.001		<0.001		0.203		<0.001
[95% CI]		[-0.028, -0.01]		[-0.026, -0.010]		[-196, 919]		[0.24, 0.9]
**HippoDeep**								
mean, std	0.940, 0.076	0.964, 0.029	0.896, 0.078	0.919, 0.025	0.147, 0.300	0.087, 0.400	3.05, 2.20	2.39, 1.30
p-value		<0.001		<0.001		<0.001		<0.001
[95% CI]		[-0.03, -0.018]		[-0.029, -0.017]		[0.035, 0.084]		[0.46, 0.86]
**FastSurfer**								
mean, std	0.964, 0.076	0.979, 0.031	0.901, 0.075	0.916, 0.039	0.130, 0.162	0.100, 0.077	3.98, 2.64	3.37, 1.57
p-value		<0.001		<0.001		<0.001		<0.001
[95% CI]		[-0.021, -0.009]		[-0.021, -0.009]		[0.017, 0.042]		[0.38, 0.84]
**QuickNat**								
mean, std	0.938, 0.094	0.939, 0.078	0.917, 0.095	0.924, 0.078	0.111, 0.241	0.084, 0.126	3.78, 4.17	2.65, 2.04
p-value		0.74		0.005		<0.001		<0.001
[95% CI]		[-0.0056, 0.004]		[-0.012, -0.002]		[0.011, 0.040]		[0.79, 1.5]
**AssemblyNet**								
mean, std	0.936, 0.030	0.941, 0.025	0.884, 0.031	0.89, 0.021	0.131, 0.052	0.122, 0.030	3.40, 1.50	3.26, 0.96
p-value		<0.001		<0.001		<0.001		0.03
[95% CI]		[-0.008, -0.003]		[-0.008, -0.003]		[0.005, 0.014]		[0.014, 0.28]

Supplementary Table S4. *Summary of the Evaluation metrics in the "best" subset in relation to the manual segmentation (n=30 patients).*

Algorithms	Failed (n)	Volumetric Similarity (mean±std)	DICE score (mean±std)	Average Hausdorff Distance (mean±std)	Hausdorff Distance (mean±std)
**e2dhipseg**	1 (2%)	0.898 ± 0.044	0.864 ± 0.038	0.142 ± 0.046	3.78 ± 4.31
**HippMapp3r**	4 (7%)	0.795 ± 0.207	0.747 ± 0.230	1.780 ± 6.590	8.53 ± 12.7
**HippoDeep**	**0 (0%)**	0.963 ± 0.062	0.909 ± 0.057	0.108 ± 0.175	3.17 ± 4.77
**FastSurfer**	**0 (0%)**	0.958 ± 0.057	0.895 ± 0.056	0.122 ± 0.087	4.09 ± 4.41
**QuickNat**	**0 (0%)**	0.939 ± 0.138	0.888 ± 0.133	0.175 ± 0.399	4.40 ± 5.15
**AssemblyNet**	**0 (0%)**	0.958 ± 0.025	0.874 ± 0.024	0.140 ± 0.039	3.67 ± 2.76
**FreeSurfer**	**3 (5%)**	0.956 ± 0.062	0.846 ± 0.130	0.473 ± 2.290	3.97 ± 4.26
**STAPLE**	**0 (0%)**	0.979 ± 0.016	0.979 ± 0.017	0.023 ± 0.024	2.11 ± 3.77

Supplementary Figure S1: 3D rendering of the hippocampus with overlaying STAPLE segmentation for one representative example. Colors were used in concordance with Fig. 3 of the main manuscript, blue for e2dhipseg, red for HippMapp3r, green for Hippodeep, light blue for FastSurfer, purple for QuickNat, and tan for AssemblyNet.

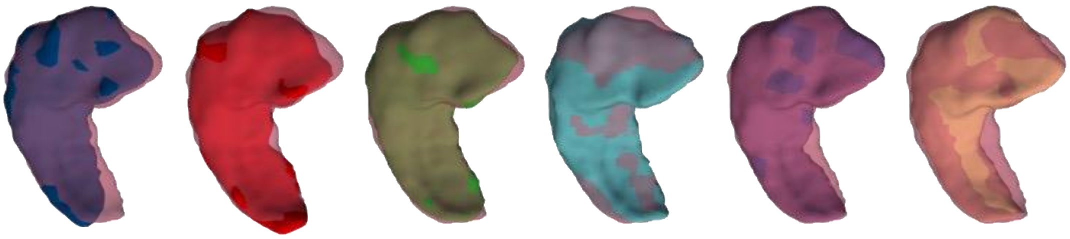

Supplementary Figure S2: Instance-based similarity classification for similarity ranks to the manual segmentation for the subset. Equal values were both assigned to the inferior category to avoid additional intermediary categories. Bark blue color with the highest similarity to STAPLE mask, red color with the lowest similarity.

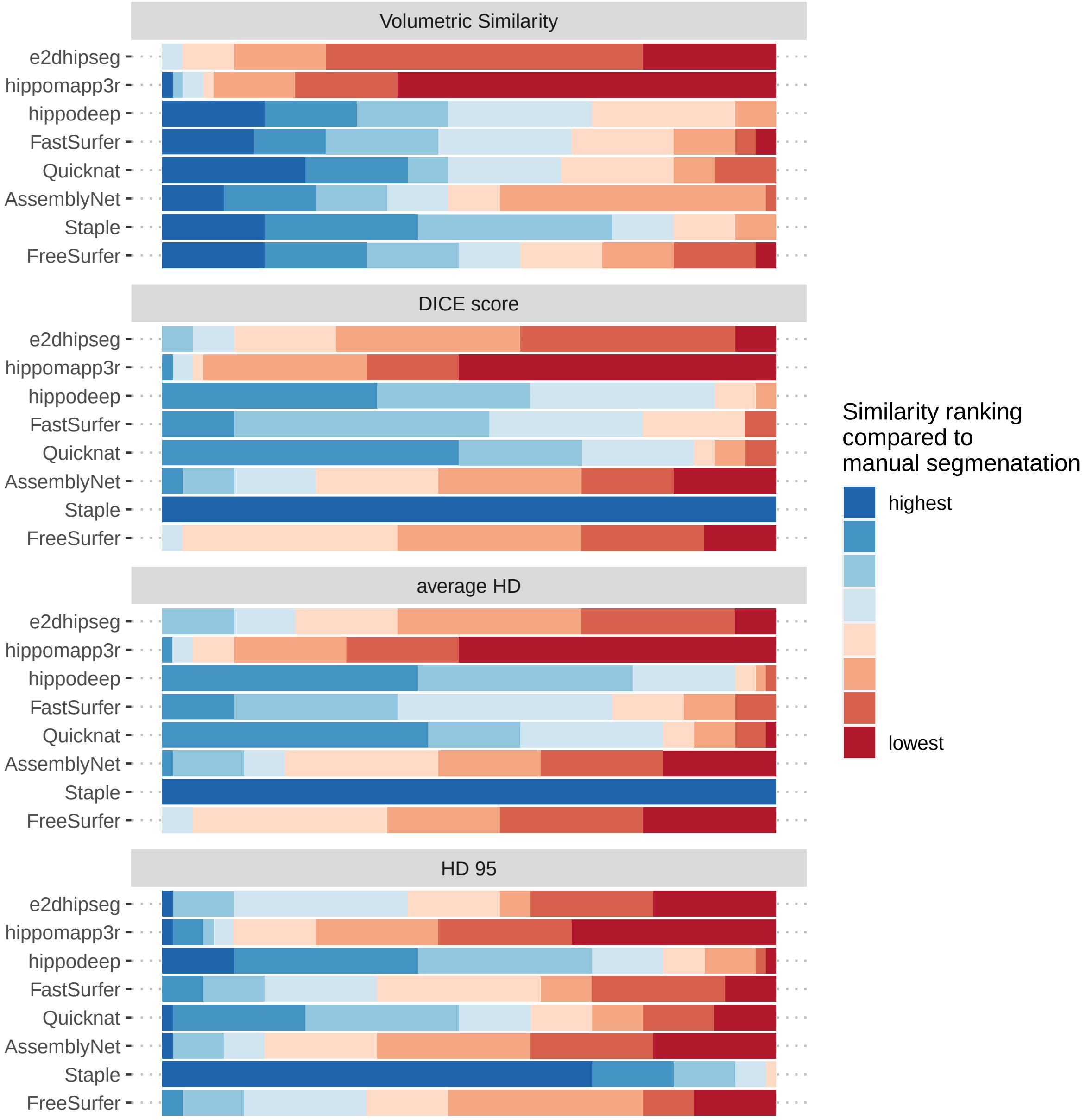

Supplement: Supplementary file 1 — Supplementary Information. [file 41598_2023_38833_MOESM1_ESM.pdf]
